# Impact of Pre-lockdown Hyper-energy on Mood and Rhythm Dysregulation in Older Adults During the COVID-19 Pandemic

**DOI:** 10.2174/0117450179344148250206065104

**Published:** 2025-02-12

**Authors:** Diego Primavera, Goce Kalcev, Fabrizio Bert, Elisa Cantone, Alessandra Perra, Massimo Tusconi, Samantha Pinna, Germano Orrù, Alessandra Scano, Enzo Tramontano, Ivan Barbov, Marcello Nonnis, Antonio Egidio Nardi, Giulia Cossu, Federica Sancassiani, Mauro Giovanni Carta

**Affiliations:** 1 Department of Medical Sciences and Public Health, University of Cagliari, Cagliari, Italy; 2 Department of Public Health Sciences, University of Turin, Turin, Italy; 3 Collegium Medicum, University of Social Sciences, Lodz, Poland; 4 University Hospital of Cagliari, Cagliari, Italy; 5 Department of Surgical Sciences, University of Cagliari, Cagliari, Italy; 6 Department of Life and Environmental Sciences, University of Cagliari, Cagliari, Italy; 7 University Clinic of Neurology, Skopje, 1000, Skopje, North Macedonia; 8 Department of Pedagogy, Psychology, Philosophy, University of Cagliari, Cagliari, Italy; 9 Institute of Psychiatry (Ipub), Federal University of Rio De Janeiro (Ufrj), Rio de Janeiro Brazil

**Keywords:** Hyper-energy, Rhythm dysregulation, Advanced technologies laboratory, Depressive symptoms, COVID-19, Bipolar spectrum, Stress

## Abstract

**Objective:**

The aim of this work is to verify whether a cohort of elderly people with hyper-energy tended to increase depressive symptoms and misaligned social and personal rhythms during the lockdown compared to a cohort of older adults without hyper-energy one year before the lockdown.

**Methods:**

The two cohorts were evaluated in April 2019 (T0) and in April 2020 (T1). Hyper-energy, cognitive performance, depressive symptoms, and social and personal rhythms were evaluated at T0 and T1.

**Results:**

In the measure of the Brief Social Rhythm Scale (BSRS) score, the differences between groups in the two observation times reach statistical significance. The sub-group with previous hyper-energy at T0 but no longer having hyper-energy at T1 increases the score by more than 5 points (a higher score indicates greater rhythm dysregulation, thus having a worse regulation of rhythms at T1), while in those individuals who didn’t have hyper-energy, the score remains substantially unchanged (+0.06). However, if the increase in the score from T0 and T1 is measured, both groups with hyper-energy at T0 presented a greater mean increase compared to people who did not have hyper-energy at T1. In the total of the eleven elderly people with hyper-energy at T0, the mean increase in BSRS score was 1.05±1.19 versus 0.06±0.98 (F=9.407, P=0.003), and in people who no longer had hyper-energy at T1, it was 1.05±1.19 versus 5.50±3.83 (F=105.0, P<0.0001). In people with hyper-energy at T0, the mean increase in the Patient Health Questionnaire-9 (PHQ-9) score was 0.72±0.75 versus 0.01±0.28 (F=37.153, P<0.0001). The gain was even higher in people who no longer had hyper-energy at T1, 1.38±1.03 *vs*. 0.01±0.28 (F=87.386, P<0.0001). An inverse linear correlation was found between energy perception (measured as the score of Item 10 of SF-12) and the score of PHQ-9 measuring depressive symptoms both at T0 and, more strongly, at T1, as well as with the BSRS scores, but only at T1.

**Conclusion:**

The study, despite the limitations of a small sample, seems to confirm a greater vulnerability to the lockdown situation in people with hyperactivity, even in the absence of psychopathology (i.e., part of the bipolar spectrum).

## 1 INTRODUCTION

The COVID-19 pandemic lockdown has placed individuals in a sort of exceptional environmental assessment, altering several risk factors for mood disorders and, among these, and not least for relevance, social and individual rhythms [1-3]. The dysregulation of individual and personal rhythms (in close connection with biorhythms) has been seen to be an element associated with mood disorders, in particular with the spectrum of bipolar disorders [4, 5]. In fact, according to some researchers, the alterations in biorhythms are considered to have a role in the genesis of mood disorders [6-8]. Also, there is evidence of a vulnerability in bipolar disorders toward triggers capable of altering social rhythms and biorhythms, such as light stimuli and light pollution [9, 10]. The circadian rhythmicity is under the control of the biological clock structures that manage the functioning of human beings under 24-hour cycles. Internal and external signals influence this clock, but light is the most important one. Socio-economic and cultural factors in modern life have led to a revolution in light patterns, which may be a cause for the increase in bipolar disorders in our society or, at least, a paradoxical stability for a strongly disadvantaging disease, and it may determine an adaptive condition of certain “subthreshold” forms [4]. A recent case-control study found that older adults with a depressive episode during the lockdown but not depressed a year earlier experienced a paradoxical increase in energy in the same year before both the lockdown and the depressive episode [11].

It was also found that non-pathological personality characteristics and traits of hyper-energy / hyperactivity (without any diagnoses of mood disorders) frequently share genetic variants that have been associated with bipolar disorder [12]. In other words, those genetic characteristics would not have been typical of bipolar disorder in “itself” but of hyper-energy, hyperactivity, and novelty-seeking traits, including people with the disorder as a sort of “tip of the iceberg” [13, 14]. Therefore, the possible onset of bipolar disorder could be understood as the convergence of those genetic variants of hyper-energy and hyperactivity with some kind of stress condition [4, 15]. It needs to be explored how factors such as different genetically specific vulnerabilities to stress, as well as the epigenetic transmission of the trauma memory and perinatal trauma, characteristics intrinsic to stress, or other environmental variables, can play a trigger for an episode of pathological mood (in the case of the predisposition to hyper-energy, this is a path that requires entirely to be followed) [16-23]. By reversing the observation perspective of the previously cited study [11], it could be interesting to observe, according to a prospective model, how elderly people with previously assessed hyper-energy/hyperactivity were coping with the lockdown (based on their mood, personal, and social rhythms) compared to elderly people who did not show this hyper-energy or hyperactivity one year earlier, before the first peak of the pandemic.

The aim of this work is to study whether the members of a cohort of elderly people with hyperactivity/hyper-energy characteristics tended to increase depressive symptoms and regulate social and personal rhythms during the lockdown with a different profile compared to a cohort of older adults without hyperactivity / hyper-energy one year before.

A parallel evaluation was conducted to verify whether the presence of hyperactivity correlated similarly with the observed variables at the two different time points.

## 2 METHODS

### 2.1 Design and Study Sample

A cohort study was performed at the end of an RCT (Randomized-Controlled Trial) [24]. This study is a secondary analysis, meaning it utilizes data originally collected for a different primary research purpose. Specifically, the data were initially gathered for a Randomized Controlled Trial (RCT) assessing the efficacy of moderate physical exercise in elderly participants. For this study, we conducted a separate analysis on this pre-existing dataset to explore the relationship between hyper-energy and mood dysregulation during the COVID-19 lockdown. By reanalyzing the data with a different focus, we aimed to generate new insights beyond the original scope of the RCT. The study was conceived to assess, as a primary outcome, the efficacy of moderate physical exercise training. This cohort study also included the individuals selected for participation in the RCT but not involved in the final sample after the signed consensus due to over-numbering the cells of randomization. They remained on a waiting list because another trial was planned but not carried out in spite of the pandemic. As described in more detail in the previous paper [24], the criteria for inclusion included: no limitation by gender, age ≥ 65 years, living at home, and positive medical evaluation for non-competitive physical activity. On the other hand, the exclusion criteria were: age less than 65 years old, presence of health conditions causing unsuitability for moderate physical activity, living in sheltered houses or assisted residences, BMI (body mass index) > 35, history of psychosis, and presence of severe brain disease.

The sample was evaluated at the entry of RCT in April 2019 (coinciding with the entry of this cohort (T0)) and in April 2020 (end of cohort (T1) serendipity during the lockdown). Hyper-energy, cognitive performance, depressive symptoms, and social and personal rhythms were evaluated at T0 and T1.

### 2.2 Instruments

In this study, as a measure of hyper-energy, we adopted item number 10 of the Short Form Health Survey in the version of 12 items (SF-12) [25] questionnaire, *“How long in the last 4 weeks did you feel full of energy?.”* Only the answer number 6 on a scale from one to six (coded “always”) was taken into consideration as indicative of hyper-energy.

The self-administered tool Patient Health Questionnaire in the version with 9 items (PHQ-9) was adopted for measuring depressive symptoms [26]. The overall score of this scale is the sum of the scores of each of the items that indicate the core symptoms of the depressive episode according to the Diagnostic and Statistical Manual of Mental Disorders (DSM-5) [27].

The functionality of social and behavioral rhythms was measured by the Italian version of the Brief Social Rhythms Scale (BSRS) [28]. A high total score on this scale indicates worse functioning.

### 2.3 Data Analysis Section

The data were analyzed using the SPSS software (version 23). The comparison between means and standard deviations of numerical data between the two cohorts at the start and end of the follow-up was conducted by means of one-way ANOVA statistics with Bonferroni corrections. The comparison at each observation point (T0 and T1) was conducted between those individuals who did not have hyper-energy at T0 (who didn't answer “6” to item 10 of the SF-12) and those who had hyper-energy at T0 (who had responded “6” to item 10 of the SF-12), and among these were also the elderly people who had hyper-energy at T0 but had no more at T1. The nominal variables were compared by the Fisher Exact test. The correlation at TO and T1 between variables was measured by Pearson’s correlation test.

In this study, hyper-energy was defined as a self-reported perception of feeling “always full of energy,” corresponding to a score of “6” on the SF-12 questionnaire item. This threshold was selected based on the study's aim to identify individuals exhibiting unusually high, sustained energy levels, a trait hypothesized to correlate with dysregulation in mood and social rhythms. While consistently high energy levels may indicate a degree of hyperactivity or heightened arousal, this classification does not imply a diagnosis of hyperactivity disorder. Instead, it aligns with emerging evidence linking such traits to mood regulation challenges, particularly under stressors like lockdown conditions. Future studies could explore a more nuanced scale to differentiate between normative high energy and potentially maladaptive hyper-energy.

### 2.4 Ethical Aspect

The study is registered on ClinicalTrials.gov (NCT03858114). The Regional Ethical Committee has approved the study with reference number PG/2018/15546, approved 25 October 2018. This study forms part of a larger research project initially designed to evaluate the effects of moderate physical exercise on elderly participants. As part of this broader project, the data used in this secondary analysis were collected during the study period and included information relevant to physical and mental health. Although the current analysis was not initially outlined in the primary study objectives, participants were fully informed during the consent process that their data might be used for future analyses within the scope of health and wellness research. The study received ethical approval for secondary analyses related to the collected data, ensuring participants were aware of the possibility of additional exploratory studies. Written informed consent was requested and obtained from each participant before their involvement in the study.

## 3 RESULTS

Table **1** shows the two cohorts: people with hyper-energy at T0 (N=11) and people without hyper-energy at T0 (N=82). The two groups were similar by sex distribution (males, where 45.5% were with hyper-energy and 46.34% without, Fisher exact test p=0.644) and age (71.81±2.91 years old referred for the people with hyper-energy and 72.68±5.03 for the people without, ANOVA 1,91df, F=0.313, p=0.577). At the end of observation during the lockdown, 6 people still had hyper-energy, of which 3 were the same as at T0.

In the measure of BSRS score (regulation of social rhythms), the differences between groups in the two observation times reach statistical significance (Table **2**). The sub-group with previous hyper-energy at T0 but no longer having hyper-energy at T1 increases the score by more than 5 points (a higher score indicates greater rhythm dysregulation, thus having a worse regulation of rhythms at T1), while in those individuals who did not have hyper-energy, the score remains substantially unchanged (+0.06). In this case, the difference did not reach statistical significance at T1 in comparison with people without hyper-energy at T0 (Table **2**). However, if the increase in the score from T0 and T1 is measured, both groups with hyper-energy at T0 presented a greater mean increase compared to people who did not have hyper-energy at T1. In the total of the eleven elderly people with hyper-energy at T0, the mean increase in BSRS score was 1.05±1.19 versus 0.06±0.98 (F=9.407, P=0.003), and in people who no longer had hyper-energy at T1, it was 1.05±1.19 versus 5.50±3.83 (F=105.0, P<0.0001).

**Graphical representation**:

At the entry of the cohort (T0), people with hyper-energy at T0 showed lower scores in the PHQ-9 scale (symptoms of depression) in comparison to old adults without hyper-energy at T0 (0.37±0.65 *vs* 2.50±3.36, ANOVA 1,91df, F=3.970 P=0.049). Furthermore, the same but with a strong significance is shown when comparing people with hyper-energy at T0, losing hyper-energy at T1 (0.12±0.33 *vs* 2.50±3.36, ANOVA 1,89 df, F=4.359; P=0.040). If the increase in the PHQ-9 score from T0 and T1 is measured, both groups with hyper-energy at T0 presented a greater mean increase compared to people who did not have hyper-energy at T1. In the total of the eleven elderly people with hyper-energy at T0, the mean increase in (PHQ-9) score was 0.72±0.75 versus 0.01±0.28 (F=37.153, P<0.0001). In people who no longer had hyper-energy at T1, the difference was 0.72±0.75 versus 1.38±1.03 (F=87.386, P<0.0001) (Table **2**).

Table **3** shows that in the overall sample, an inverse linear correlation was found between energy perception (measured as the score of Item 10 of SF-12) and the score of PHQ-9 measuring depressive symptoms both at T0 (R=-0.345, p<0.001) and more stronger, at T1 (R=-0.539, p<0.001). As far as the relationship between energy perception measured as the score of Item 10 of SF-12 and the score of BSRS, an inverse linear correlation was found only at T1 during the lockdown (R=-0.235, p=0.025). Thus, the higher the energy perception score, the more dysregulated the social and behavioral rhythms are (a high BSRS score indicates dysregulated rhythms). However, it should be noted that this relationship is not strong.

Finally, the score relating to rhythm dysregulation is directly correlated to the extent of depressive symptoms measured as a PHQ-9 score both at the first assessment (R=0.322, p=0.001) and with more consistency during the lockdown (R=0.366, p<0.001). This could be caused by the specific response to the lockdown and the possible dysregulation of social rhythms linked to the lockdown.

1. **BSRS Scores**: This line chart displays BSRS scores at T0 and T1 for each group (without hyper-energy, with hyper-energy, and with hyper-energy lost at T1), showing changes in rhythm dysregulation over time.

2. **PHQ-9 Scores**: This chart illustrates PHQ-9 scores for the same groups at T0 and T1, highlighting variations in depressive symptoms across the different conditions.

**Fig. (1):**
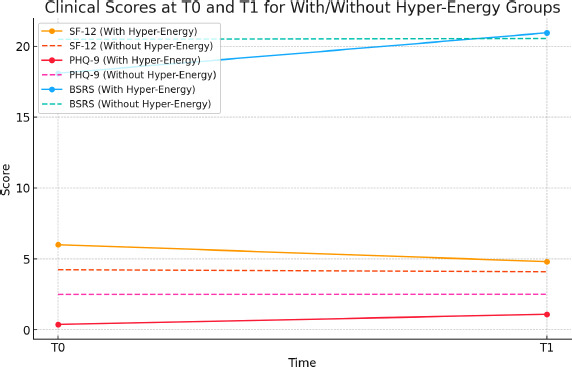
Clinical scores at T0 and T1 for with/without hyper-energy groups.

These visualizations provide a clearer comparison of BSRS and PHQ-9 score changes between groups, enhancing the interpretation of the data. (Fig. **1**)

The correlation analysis indicates:

1. SF-12 and PHQ-9 at T0: Correlation of -1.0, indicating a strong inverse relationship between energy perception (SF-12) and depressive symptoms (PHQ-9) at baseline.

2. SF-12 and BSRS at T0: Correlation of -1.0, suggesting a strong inverse relationship between energy perception and rhythm dysregulation (BSRS) at baseline.

3. SF-12 and PHQ-9 at T1: Correlation of -1.0, again showing a strong inverse relationship during the lockdown.

4. SF-12 and BSRS at T1: Correlation of 1.0, indicating a direct relationship between energy perception and rhythm dysregulation during the lockdown.

These correlations suggest that as energy perception increases, depressive symptoms and rhythm dysregulation generally decrease at both time points, except for the direct relationship with BSRS at T1, potentially reflecting unique lockdown-related impacts on rhythm dysregulation.

## 4 DISCUSSION

The study demonstrates that a sample of elderly people who perceived themselves to be “always full of energy” one year before the lockdown showed a greater increase in depressive symptoms during the lockdown than the parallel increase in a sample of old adults without this perception of hyper-energy. Considering only the sub-sample of older adults who felt full of energy at the first assessment but lost this perception during the lockdown (while three individuals maintained it), this sub-sample also showed a significant loss in the regulation of social and behavioral rhythms compared to the control cohort, those who did not respond with a '6' to Item 10 of the SF-12 questionnaire at the first assessment, and therefore did not feel 'always full of energy. The study also confirmed that there is a direct correlation between dysregulation of social and personal rhythms and depressive symptoms (a direct correlation between PHQ-9 and BSRS scores) and an inverse correlation between depressive symptoms and perception of energy (the lower the perception of the highest personal energy, the more depressive symptoms there are). It was also found that there is an inverse link between rhythm dysregulation and energy (the higher the energy, the lower the rhythm regulation score). The trend highlighted by people who had hyper-energy before the lockdown (those who lost the most energy in the entire sample of elderly people and those who increased their depressive symptoms) is in line with the highlighted correlations. Those who have lost hyper-energy also have a greater loss of rhythm regulation. However, it remains to be explained why those who had a lot of energy before the lockdown lost, on average (much) more energy and increased depressive symptoms than others during the lockdown. Also, considering, in fact, that they were doing very well.

Recently, the issues have received more attention as the lockdown determined by the COVID-19 pandemic may have impacted social rhythms in people with a vulnerability to mood disorders,specifically in the elderly [29]. Old adults are, in fact, expected to suffer from changes in social rhythms and related biorhythms more than young people. Moreover, it should be considered that there might be significant differences between elderly people living in high-density urban centers and those living in rural communities during the lockdown, especially among the most vulnerable population groups, who face conditions that significantly disrupt biorhythms, and who have comorbidities, often typical of the elderly population [30-34]. Concerning sleep, they wake up and go to sleep earlier than younger adults [35]. Their sleep is interrupted by more awakenings, and their sleep pattern shows shorter rapid eye movement [36]. Interestingly, the loss of sleep during human life is calculated at around 3 minutes every year for people over 40 years old [37]. Research on jet lag found that difficulties in adapting to rhythms increase with age [38]. In addition to the specific vulnerability relating to the impact of the lockdown, old adults were exposed to a greater risk of death if exposed to COVID. They would also have suffered from fear, stress, and greater restrictions on social relationships during the lockdown [39]. Unexpectedly, several studies found a low risk of stress and depressive disorders in several countries during the pandemic [40, 41].

The explanation for this worsening (which, however, does not reach pathology) in healthy and hyperactive people before the lockdown can arise from the concept of bipolar spectrum in the neo-Kraepelinian meaning of the term and in the evolutionary vision of the concept of bipolar spectrum [13, 42-48]. From the social point of view, if we hypothesize that these hyperactive, healthy, and well-integrated old adults represent the non-pathological sub-stratum of the bipolar spectrum, perhaps we can understand their responses to this particular situation, keeping in mind what is known about the responses that, in similar situations, have been done by people with bipolar disorder. It is well known that the dysregulation of biological and social rhythms is a recognized risk factor for all mood disorders [49-51], but it is also acknowledged that this element represents a stronger vulnerability factor and, according to some, an intrinsic characteristic of bipolar disorders [52-54], playing even a relevant role in the onset of bipolar disorder [55]. From this perspective, it may be useful to remember that in cohorts of patients with bipolar disorder subjected to a different level of lockdown severity, those who were in Italy (therefore under a very strict lockdown) had many more relapses than those who were in Tunisia (under a lighter lockdown) [2].

This study has several limitations that may impact the generalizability of its findings. First, the small sample size of elderly participants identified with hyper-energy traits limits the power of the statistical analyses and may introduce bias, reducing the precision of the observed associations. Future research should aim for larger sample sizes to validate these findings. Additionally, COVID-19 infection, whether symptomatic or asymptomatic, was not included as a covariate in this analysis. Given the potential neurological and psychological effects of COVID-19, undetected or unaccounted infections could have influenced mood and energy regulation in participants. Including COVID-19 status as a covariate in future studies could help clarify its role in mood and rhythm dysregulation among the elderly during the pandemic. While the limitation of this work is the result of a secondary analysis on a database that was collected and did not have the same main objectives as those of this specific study, the particular and unexpected coincidence of the last evaluation with the lockdown made it of interest. Therefore, the considerations that the study produces have an eminent heuristic value.

## CONCLUSION

This study suggests that individuals aged 65 and older who exhibit signs of hyperactivity or consistently high energy levels may be more susceptible to depressive symptoms under conditions of social restriction, such as those experienced during the COVID-19 lockdown. The findings indicate that elevated energy perception before lockdown was associated with greater rhythm dysregulation and an increase in depressive symptoms during the restrictive period. Recognizing this vulnerability could inform targeted mental health support for older adults with hyperactive traits in future lockdown scenarios or other prolonged social restrictions.

## Figures and Tables

**Figure:**
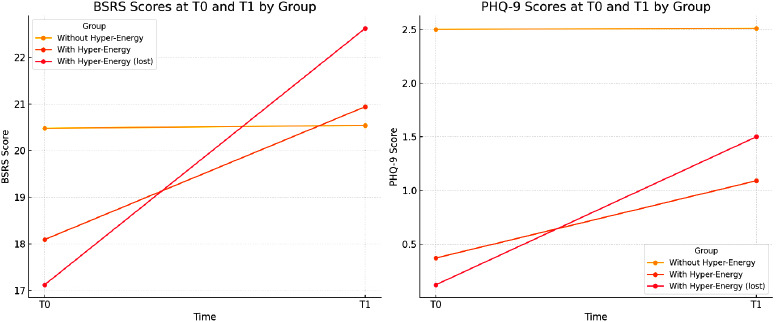


**Table 1:** Characteristics of the study sample.

	**Hyper-Energy** **T0 (N=11)**	**Without** **Hyperenergy T0 (N=82)**	Comparison (with hyper-energy versus without hyper-energy)*	**Total** **(N=93)**
**Hyper-energy** **T1**	6 (3 from T0)	87		(Frequency of people with Hyper-energy T0 *vs* T1 Fisher exact test p=0.135*)
**Male**	5 (45.5%)	38 (46.34%)	Fisher exact test p=0.644	43 (46.2%)
**Age**	71.81±2.91	72.68±5.03	ANOVA 1,91df F=0.313 P=0.577	72.58±4.78

**Table 2:** Comparison of people with and without hyper-energy at T0 concerning social rhythms and depressive symptoms.

	**Without hyper-energy at T0 (N=82)** **(Pivot)**	**With** **hyper-energy at T0** **(N=11)**	**ANOVA 1 way (1,91 df)**	**With** **hyper-energy at T0, but losing hyper-energy** **at T1** **(N=8)**	**ANOVA 1 way (1,89 df)**	**Total** **(N=93)**
**BSRS Score T0**	20.48±8.50	18.09±5.35	F=0.821 P=0.367	17.12±5.96	F=0.472 P=0.494	20.21 ± 8.13
**BSRS Score T1**	20.54±7.20	20.94±9.03	F=0.028 P=0.867	22.62±7.31	F=1.687 P=0.197	20.90 ± 8.82
**Difference** **T0-T1**	0.06±0.98	1.05±1.19	F=9.407 P=0.003	5.50±3.83	F=105.0 P<0.0001	0.69±0.69
**PHQ-9 T0**	2.50±3.36	0.37±0.65	F=3.970 P=0.049	0.12±0.33	F=4.359; P=0.040	2.25±3.04
**PHQ-9 T1**	2.51±3.27	1.09±1.35	F=1.613 P=0.207	1.5±2.64	F=0.577 P=0.449	2.34± 3.49
**Difference** **T0-T1**	0.01±0.28	0.72±0.75	F=37.153 P<0.001	1.38±1.03	F=87.386 P<0.0001	
**SF-10 T0**	4.24±1.28	6.0±0.0	F=20.601 P<0.0001	6.0±0.0	F=14.171 P<0.0001	4.24±1.13
**SF-10 T1**	4.09±1.08	4.81±1.19	F=4.212 P=0.043	4.14±0.98	F=0.016 P=0.900	4.18±1.09
**Difference** **TO-T1**	0.16±0.10	1.19±0.55	F=244.16 P<0.0001	1.86±0.75	F=633.0 P<0.0001	

**Table 3:** Correlation between energy perception (Item 10 of SF-12) with depressive symptoms (PHQ-9) and social rhythms (BSRS).

	**PHQ-9 R**	**PHQ-9 R**	**BSRS R**	**BSRS**
**T0 SF12 (10) N=93**	-0.345	p<0.001	-0.162	P=0.321
**T1 SF12 (10) N=93**	-0.539	p<0.001	-0.235	P=0.025
**T0 BSRS N=93**	0.322	P=0.01	-	-
**T1 BSRS N=93**	0.366	p<0.001	-	-

## Data Availability

The data sets used and/or analysed during this study are available from the corresponding author [G.K] upon request.

## LIST OF ABBREVIATION

BMI: = Body Mass Index
RCT: = Randomized Controlled Trial
BSRS: = Brief Social Rhythms Scale

## STANDARDS OF REPORTING

STROBE guidelines were followed.
